# LIN-32/Atonal Controls Oxygen Sensing Neuron Development in *Caenorhabditis elegans*

**DOI:** 10.1038/s41598-017-07876-4

**Published:** 2017-08-04

**Authors:** Teresa Rojo Romanos, David Pladevall-Morera, Kasper Langebeck-Jensen, Stine Hansen, Leelee Ng, Roger Pocock

**Affiliations:** 1 Development and Stem Cells Program, Monash Biomedicine Discovery Institute and Department of Anatomy and Developmental Biology, Monash University, Melbourne, Victoria 3800 Australia; 2 Biotech Research and Innovation Centre, University of Copenhagen, Ole Maaløes Vej 5, Copenhagen, Denmark

## Abstract

Development of complex nervous systems requires precisely controlled neurogenesis. The generation and specification of neurons occur through the transcriptional and post-transcriptional control of complex regulatory networks. In vertebrates and invertebrates, the proneural basic-helix-loop-helix (bHLH) family of transcription factors has multiple functions in neurogenesis. Here, we identified the LIN-32/Atonal bHLH transcription factor as a key regulator of URXL/R oxygen-sensing neuron development in *Caenorhabditis elegans*. When LIN-32/Atonal expression is lost, the expression of URX specification and terminal differentiation genes is abrogated. As such, *lin*-*32* mutant animals are unable to respond to increases in environmental oxygen. The URX neurons are generated from a branch of the cell lineage that also produces the CEPDL/R and URADL/R neurons. We found development of these neurons is also defective, suggesting that LIN-32/Atonal regulates neuronal development of the entire lineage. Finally, our results show that aspects of URX neuronal fate are partially restored in *lin*-*32* mutant animals when the apoptosis pathway is inhibited. This suggests that, as in other organisms, LIN-32/Atonal regulates neuronal apoptosis.

## Introduction

The development of complex systems like the brain requires the generation of a vast array of cell types with distinct morphology and function. Such cellular diversity is achieved by transcription factors and microRNAs that regulate the generation and specification of terminal neuronal subtypes^1,2^. Previous studies using model organisms have shown that a family of basic-helix-loop-helix transcription factors act as proneural regulators to control the development of neuroectodermal progenitor cells^3^. Proneural genes were first identified in *Drosophila* in the 1980s and have subsequently been shown to have multiple functions in the developing brain, including neuronal specification and guidance^4–6^. Atonal is a well-studied member of the proneural basic-helix-loop-helix transcription factor family that was originally identified in *Drosophila*, where it is required for correct development of sensory organ precursors^7^. The Atonal homolog in mouse (Atoh1) is also expressed in the developing nervous system where it performs conserved functions in the development of the cochlea and the specification of auditory hair cells^8–10^. Multiple studies have shown catastrophic apoptosis in the cochlea of Atoh1-null mouse embryos, leading to hearing loss^8,10,11^. In *C*. *elegans*, the Atonal homolog LIN-32 is required for the development of multiple neuronal lineages that generate sensory rays within the male reproductory organ^12,13^, mechanosensory neurons^14,15^, Q neuroblasts^16^ and the CEPD and PDE dopaminergic neurons^17^.

We study the URXL/R body cavity neurons in *C*. *elegans* as a model for neuronal development. These neurons regulate multiple aspects of animal behaviour and physiology^18,19^. The cell bodies and ciliated dendrites of the URX neurons are positioned within the coelomic fluid^20^, which potentially enable these neurons to receive and transmit information systemically. A major function of the URX neurons is in oxygen sensing^18^. Through expression of the soluble guanylate cyclases GCY-35 and GCY-36, the URX neurons coordinate behavioral responses to sensing of environmental O_2_ upshifts^18^. In addition, a recent function for the URX neurons has been identified in the integration of O_2_ availability and internal metabolic state^19^.

The specialised functions of the URX neurons are facilitated through the expression of particular terminal differentiation genes^18,19,21^. We and others previously found that expression of terminal fate reporters for URX neuron fate is regulated by the Sox transcription factor EGL-13 and the AHR-1/aryl hydrocarbon receptor^21,22^. Here, we find that LIN-32/Atonal is also required for the correct development of the URX O_2_-sensing neurons. In *lin*-*32* mutant animals, reporters for URX-expressed transcription factors, including EGL-13, and terminal differentiation genes are abrogated. As a result, *lin*-*32* mutant animals are unable to respond to O_2_ upshifts_._ We found that LIN-32 is also required for the expression of terminal fate reporters for other neurons generated from the URX sublineage (URADL/R and CEPDL/R), indicating that neuronal development within the entire sublineage is defective. Our subsequent investigation found that aspects of URX neuronal fate are restored when apoptosis is perturbed, suggesting that LIN-32 either acts to inhibit apoptosis in this lineage or that the misspecified state of these neurons promotes cell death. As such, our study has revealed a previously unappreciated function of LIN-32/Atonal in regulating O_2_-sensing neuron development in the brain.

## Results

### LIN-32/Atonal is required for URX neuron development

**Figure 1:**
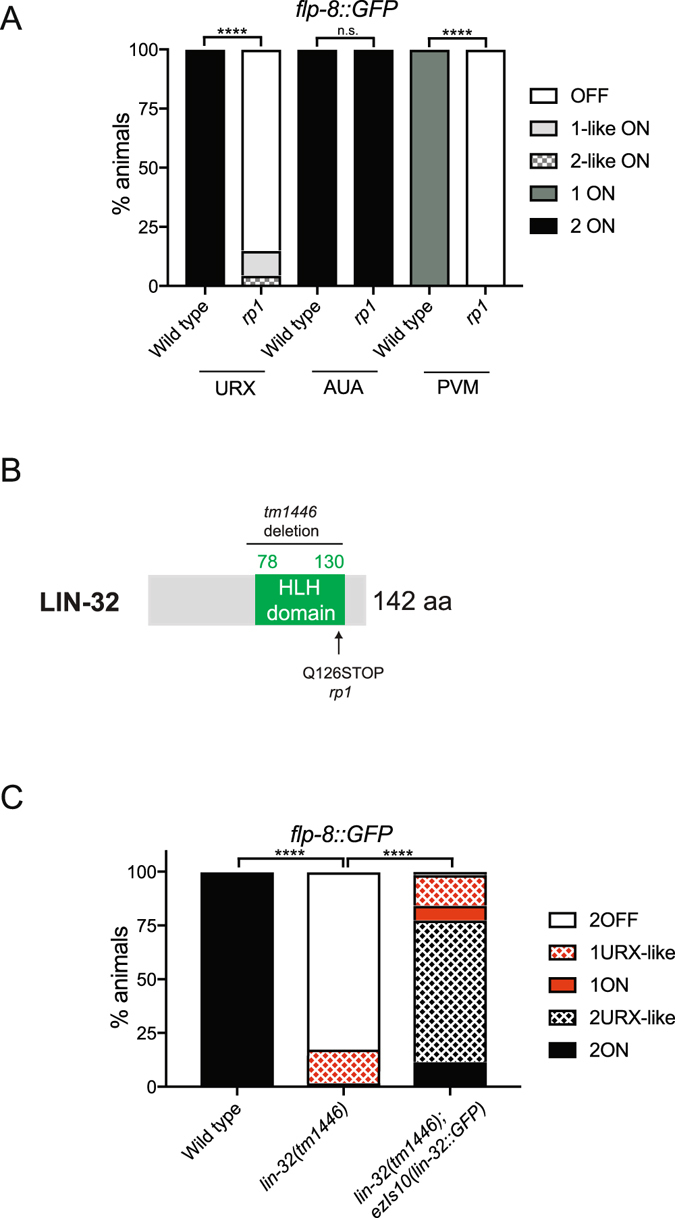
*rp1* is a lesion in *lin*-*32* and causes URX developmental defects. (**A**) Quantification of *rp1*-induced URX defects using the *flp*-*8*::*GFP* reporter of URX fate. *flp*-*8*::*GFP* is expressed in the URX, AUA and PVM neurons^23^. In *rp1* animals, expression of *flp*-*8*::*GFP* is almost abolished in the URX and PVM neurons while expression in the AUA neurons is unaffected. 1URX-like and 2URX-like indicate that the neuronal morphology is similar to URX but the neuron is positioned incorrectly. N > 60. Statistical significance between wild-type and *lin*-*32*(*rp1*) animals was evaluated using a t-test. ****P < 0.0001. (**B**) Molecular identity of the *rp1* allele and the previously described *lin*-*32*(*tm1446*) deletion allele. The *rp1* lesion is a C to T transition that converts a glutamine (Q) to a premature amber stop codon. (**C**) Quantification of *flp*-*8*::*GFP* expression in the URX neurons in wild type, *lin*-*32*(*tm1446*) mutant and *lin*-*32*(*tm1446*); *ezIs10*(*lin*-*32*::*GFP *+* unc*-*119*(+) rescued animals. N > 60. Statistical significance between wild-type and *lin*-*32*(*tm1446*) animals was evaluated using one-way ANOVA analysis. ****P < 0.0001. Note the high number of 1URX-like and 2URX-like animals in rescued animals. These are neurons that exhibit a URX morphology but are mispositioned. We found that the *lin*-*32*::*GFP* rescuing transgene can cause URX mispositioning defects in wild-type animals (not shown) suggesting that dosage of LIN-32 is important for neuron position.

In order to identify regulators required for the development of the URX O_2_-sensing neurons, we performed a classical EMS forward genetic mutagenesis screen using the URX fluorescent reporter strain *ynIs22*[*flp*-*8*::*GFP*]. This reporter is consistently expressed in the URXL/R neurons in addition to the AUAL/R and PVM neurons^23^. We isolated a mutant allele, *rp1*, which exhibits highly penetrant loss of GFP expression in both the URX and PVM neurons (Fig. 1A). We used single nucleotide polymorphism (SNP) mapping^24^ to identify the molecular lesion responsible for this defect. This analysis narrowed down the location of the lesion close to genetic position −17 on the X chromosome. Mutations in the *lin*-*32* gene, which encodes a bHLH transcription factor^13^ and is located close to this region (−16.05), had previously been described to have defects in PVM specification^15^. Therefore, we manually sequenced the *lin*-*32* locus and identified a base-pair change C > T in the *rp1* mutant, which converts a glutamine (Q) at position 126 to a premature amber STOP codon (Fig. 1B). To confirm that loss of LIN-32 causes defects in URX development, we crossed the *flp*-*8*::*GFP* fluorescent reporter with the previously described *lin*-*32*(*tm1446*) allele^25^, a 511 bp deletion that removes the coding region of the bHLH domain, and is a presumed null allele. Loss of *flp*-*8*::*GFP* expression in the URX neurons was phenocopied in the *lin*-*32*(*tm1446*) mutant strain showing that LIN-32 is important for URX development (Fig. 1C). To confirm that loss of LIN-32 is causative, *flp*-*8*::*GFP* expression was restored in the URX neurons of *lin*-*32*(*tm1446*) mutant animals through expression of an integrated *lin*-*32*::*GFP* rescuing translational transgene (*ezIs10*)^26^ (Fig. 1C).

**Figure 2:**
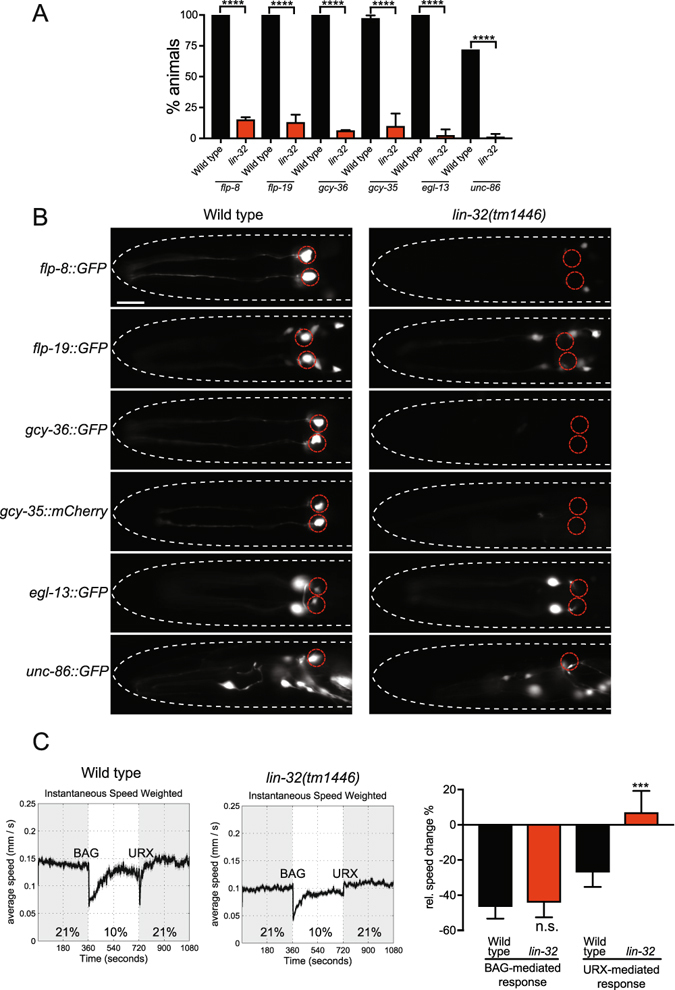
LIN-32 is required for URX specification and function. (**A**) Quantification of *lin*-*32*(*tm1446*)-induced URX defects in reporters for URX neuronal fate. Loss of *lin*-*32* severely affects the expression of all URX reporters tested: *flp*-*8*, *flp*-*19*, *gcy*-*36*, *gcy*-*35*, *egl*-*13* and *unc*-*86*. The black (wild type) and red (*lin-32*) bars represent the percentage of worms that show expression in either 1 or 2 URX neurons. 1-like and 2-like indicate that the neurons have a URX morphology but are mispositioned. N > 60. Statistical significance between wild-type and *lin*-*32*(*tm1446*) animals was evaluated using a t-test. ****P < 0.0001. (**B**) Micrographs of representative animals expressing fluorescent markers for the URX neurons (*flp*-*8*, *flp*-*19*, *gcy*-*36*, *gcy*-*35*, *egl*-*13* and *unc*-*86*) in wild type and *lin*-*32*(*tm1446*) mutant animals. URX neuron positions are marked with red dashed circles. Anterior to the left. Ventral views except for *unc*-*86*::*GFP* which is a lateral view. Scale bar 20 μm. (**C**) *lin*-*32* oxygen-sensing behavior analysis. Locomotion speed of wild type (left) and *lin*-*32*(*tm1446*) mutant animals (center) during O_2_ concentration shifts between 21% and 10%. The data represent averages of multiple assays. The right graph shows the quantification of changes in relative speed in response to changes in O_2_ concentration. *lin*-*32*(*tm1446*) mutants fail to respond to O_2_ upshifts (URX-mediated) but exhibit a similar response to wild type animals to O_2_ downshifts (BAG-mediated). Statistical significance between wild-type and *lin*-*32*(*tm1446*) animals was evaluated using one-way ANOVA analysis. ***P < 0.001; n.s., not significantly different from wild type controls. Assays were repeated at least four times using 80–120 animals per assay.

To better understand the function of *lin*-*32* in URX specification, we used fluorescent reporter strains to monitor expression of a battery of terminal genes expressed in the URX neurons (Fig. 2A,B)^21^. We analyzed the expression of the terminally-expressed guanylate cyclases GCY-35 and GCY-36, and the Phe-Met-Arg-Phe-NH2 (FMRF-amide)-related peptides FLP-8 and FLP-19. Using the *lin*-*32*(*tm1446*) allele, we found that the absence of *lin*-*32* abrogated expression of all the URX-expressed genes we tested (Fig. 2A,B). Previous studies have shown that the EGL-13/Sox and UNC-86/POU-homeodomain transcription factors control the expression of the URX terminal gene battery^21,22^. We observed that expression of the *egl*-*13*::*GFP* and *unc*-*86*::*GFP* reporters were also abrogated in *lin*-*32*(*tm1446*) mutant animals (Fig. 2A,B). Together our data indicate that LIN-32 plays a crucial role in the development of the URX neurons, genetically upstream of URX specification genes.

### *lin*-*32* mutants are defective in oxygen sensing

The URX neurons are required for sensing and coordinating responses to fluctuations of O_2_^18^. These responses are controlled by the guanylate cyclases GCY-35 and GCY-36^18^. Since LIN-32 is required for the expression of these O_2_-sensing guanylate cyclases, in addition to the URX specification transcription factors, we hypothesized that *lin*-*32* mutant animals would be defective in O_2_ sensing. We therefore used a well-established behavioral paradigm to examine the importance of LIN-32 in O_2_ sensing^18,27^. The locomotion speed of *C*. *elegans* in response to O_2_ shifts is regulated by the BAG and URX neurons. We tracked animals in a chamber without food, in an air-flow that switched between 21% to 10% O_2_ (BAG-mediated response) and from 10% to 21% O_2_ (URX-mediated response). In contrast to wild type, we found that *lin*-*32*(*tm1446*) mutant animals were unable to respond to O_2_ upshifts, whereas the ability to respond to O_2_ downshifts was unaffected (Fig. 2C). These data indicate that the O_2_-sensing function of the URX neurons is abrogated in *lin*-*32*(*tm1446*) mutant animals, likely due to the loss of guanylate cyclase expression.

### *lin*-*32* controls the development of all neurons in the URX lineage

**Figure 3:**
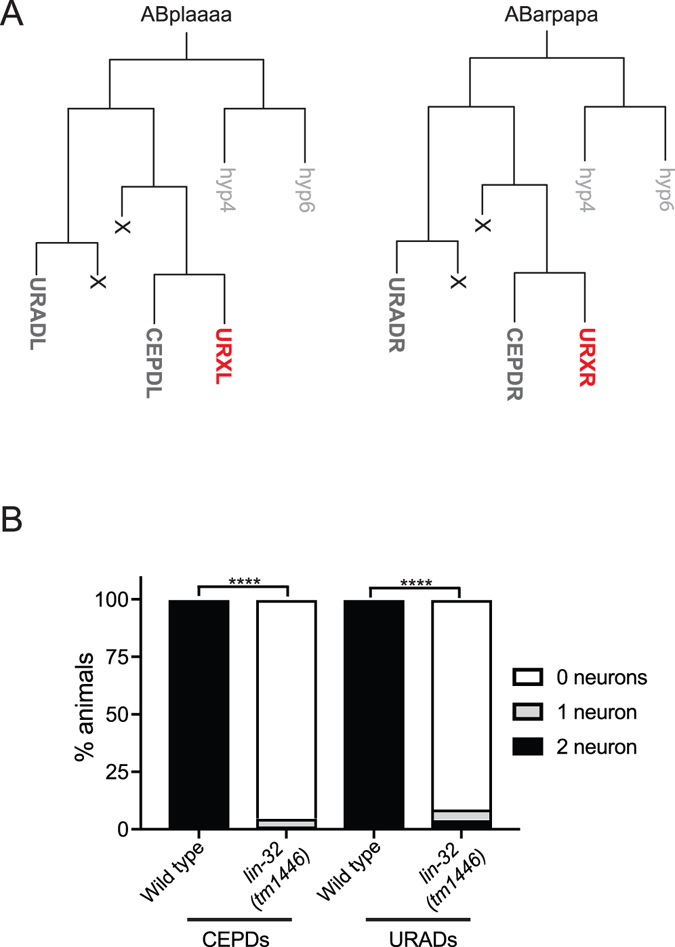
Mutations in *lin*-*32* disturbs neuronal development in the ABplaaaa and ABarpapa lineages. (**A**) Lineage diagrams of ABplaaaa and ABarpapa from which the URXL and URXR neurons are generated. ABplaaaa and ABarpapa emanate two sublineages - a posterior lineage that generates two hypodermal cells (hyp4 and hyp6) and an anterior lineage that generates the URX, CEPD and URAD neurons plus two apoptotic deaths (marked with an X). (**B**) Quantification of *dat*-*1*::*GFP* (CEPD neurons) and *flp*-*21*::*GFP* (URAD neurons) expression in wild type and *lin*-*32*(*tm1446*) mutant animals. N > 65. Statistical significance between wild-type and *lin*-*32*(*tm1446*) animals was evaluated using a t-test. ****P < 0.0001.

The URXL/R neurons are derived from the AB lineage and are sisters of the dopaminergic CEPDL/R neurons (Fig. 3A)^28^. We asked whether LIN-32 performs a specific function in the development of the URX neurons or plays a more general role in the development of other neurons closely related by lineage. To this end, we crossed *lin*-*32*(*tm1446*) mutant animals into the *vtIs1*[*dat*-*1*::*GFP*] fluorescent reporter strain, which is expressed in all dopaminergic neurons in *C*. *elegans*, including the CEPD neurons^29^. We found that the expression of this reporter is abolished in the CEPD neurons of *lin*-*32*(*tm1446*) mutant animals (Fig. 3B), confirming a previous study^17^. The URX and CEPD neurons are generated from the ABplaaaa and ABarpapa branches of the AB lineage. The other cells generated by these lineages are four hypodermal cells (two hyp4 and two hyp6 cells), the URADL/R neurons and four additional cells that undergo apoptosis^28^. We therefore asked whether LIN-32 is also required for the development the URAD neurons, which are generated slightly earlier and from an adjacent branch of the lineage to the URX neurons. We crossed a URAD neuron reporter, *ynIs80*[*flp*-*21*::*GFP*], into the *lin*-*32*(*tm1446*) mutant strain and observed that expression in the URAD neurons was completely abolished. Therefore, LIN-32 is required for the development of all neurons in this sublineage (URX, CEPD and URAD). Previous studies have shown that in the absence of LIN-32, neurons from certain lineages exhibit a hypodermal state^13^. We examined whether additional hypodermal cells are present in *lin*-*32*(*tm1446*) mutant animals using a hypodermal fluorescent reporter strain *rpIs109*[*dpy*-*7*::*NLS*::*dsRed2*]. We found that the number of hypodermal cells present in the head of *lin*-*32*(*tm1446*) mutant animals (17–24 cells) was no different from wild type (17–25 cells) in the head of L4 larvae (N > 63). This suggests that the six neurons lost from the ABplaaaa and ABarpapa lineages in *lin*-*32* mutant animals do not acquire a hypodermal fate. Taken together, these results indicate that LIN-32 is required for the development of all neurons generated from this sublineage.

### *ham*-*1* mutants exhibit defects in URX development

**Figure 4:**
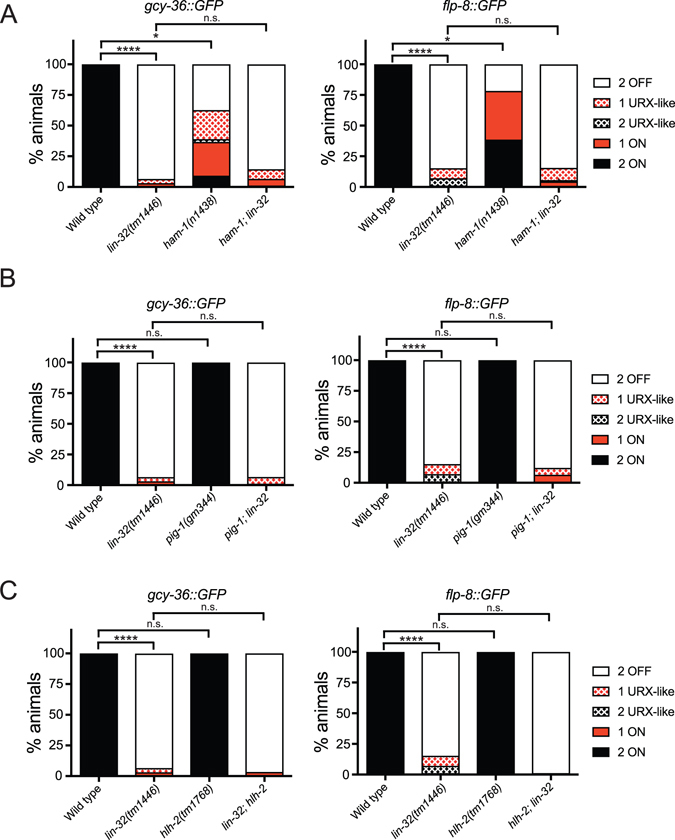
The role of HAM-1, PIG-1 and HLH-2 in URX specification. (**A**) HAM-1 regulates URX differentiation. Quantification of *gcy*-*36*::*GFP* (left) and *flp*-*8*::*GFP* (right) expression in wild type, *lin*-*32*(*tm1446*), *ham*-*1*(*n1438*) and *lin*-*32*(*tm1446*); *ham*-*1*(*n1438*) mutant animals. N > 70. (**B**) PIG-1 is not required for URX specification. Quantification of *gcy*-*36*::*GFP* (left) and *flp*-*8*::*GFP* (right) expression in wild type, *lin*-*32*(*tm1446*), *pig*-*1*(*gm344*) and *lin*-*32*(*tm1446*); *pig*-*1*(*gm344*) mutant animals. N > 70. (**C**) HLH-2 is not required for URX specification. Quantification of *gcy*-*36*::*GFP* (left) and *flp*-*8*::*GFP* (right) expression in wild type, *lin*-*32*(*tm1446*), *hlh*-*2*(*tm1768*) and *lin*-*32*(*tm1446*); *hlh*-*2*(*tm1768*) mutant animals. N = 90. Statistical significance between wild-type and mutant strains was evaluated using one-way ANOVA analysis. ****P < 0.0001; *P < 0.01; n.s. not significantly different from control.

LIN-32 has been shown to function in parallel with the storkhead transcription factor, HAM-1 (HSN abnormal migration), to regulate the migration and division of Q neuroblasts^16^. Thus, we tested whether similar means of regulation exist during URX development by crossing a *ham*-*1*(*n1438*) strong loss-of-function mutant^30^ with two URX fluorescent reporters - *kyIs417*[*gcy*-*36*::*GFP*] and *ynIs22*[*flp*-*8*::*GFP*]. We found that *ham*-*1*(*n1438*) mutant animals exhibit defects in the expression of both markers in the URX neurons (Fig. 4A). When we analyzed the *lin*-*32*(*tm1446*); *ham*-*1*(*n1438*) double mutant we observed a similar phenotype to the *lin*-*32* single mutant (Fig. 4A). Due to the high penetrant loss of URX marker expression in *lin*-*32* mutant animals, we cannot conclude whether *ham*-*1* functions in a parallel or in the same pathway as *lin*-*32*. However, we have found that *ham*-*1* is important for the development of the URX neurons.

### *pig*-*1* and *hlh*-*2* do not regulate the development of the URX neurons

It has been shown that HAM-1 promotes the expression of the serine/threonine kinase PIG-1/MELK to regulate the Q.a asymmetric division^31–33^. We therefore asked whether *pig*-*1* mutant animals exhibit similar defects in the URX neurons as the *ham*-*1* mutant. We crossed the *pig*-*1*(*gm344*) null mutant with two fluorescent reporters for the URX neurons - *kyIs417*[*gcy*-*36*::*GFP*] and *ynIs22*[*flp*-*8*::*GFP*] (Fig. 4B). We observed no detectable change in URX reporter expression in *pig*-*1*(*gm344*) mutant animals, indicating that PIG-1 is not required for proper development of the URX neurons (Fig. 4B). Furthermore, the *pig*-*1*(*gm344*); *lin*-*32*(*tm1446*) double mutant animals exhibit the *lin*-*32* single mutant URX phenotype. These results suggest that HAM-1 regulates URX development independently of PIG-1.

In other neuronal lineages, LIN-32 functions together with the bHLH transcription factor HLH-2/E/daughterless^12^. Furthermore, it has been shown that LIN-32 and HLH-2 physically interact *in vitro* ^12^. We therefore asked whether the ubiquitously expressed *hlh*-*2* also functions with *lin*-*32* to regulate URX development. To this end, we crossed the *hlh*-*2*(*tm1768*) mutant with two fluorescent reporters for the URX neurons - *kyIs417*[*gcy*-*36*::*GFP*] and *ynIs22*[*flp*-*8*::*GFP*] (Fig. 4C). We found that the *hlh*-*2*(*tm1768*) mutation does not affect the expression of these URX reporters. Taken together, these data suggest that LIN-32 acts independently of HLH-2 in the regulation of URX development.

### Suppression of apoptosis partially restores URX fate in *lin*-*32* null mutants

We have shown that in addition to the URXL/R neurons, LIN-32 regulates the expression of neuronal reporters for the two other neuronal subtypes generated from the same lineage (URADL/R and CEPDL/R) (Fig. 3). Furthermore, LIN-32 is required for the expression of two URX-fate-determining transcription factors EGL-13/Sox and UNC-86/POU. This indicates that LIN-32 functions upstream of these factors during URX development and is perhaps required for URX generation or survival. Due to the previously identified function for Atonal in *Drosophila* tumor formation^34^, we speculated that LIN-32 might regulate the programmed cell death (apoptosis) program of the URX neurons.

**Figure 5:**
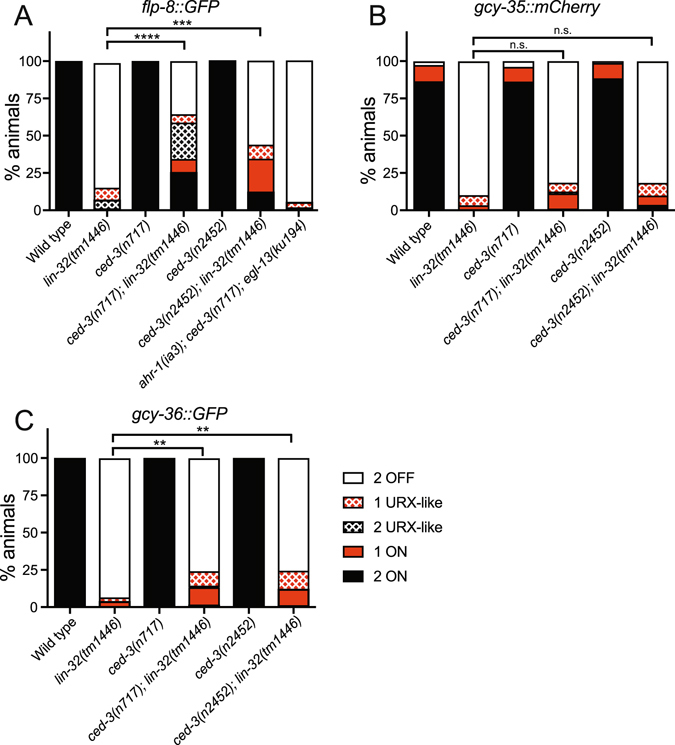
URX cell fate is restored by inhibiting apoptosis. (**A**) Quantification of *flp*-*8*::*GFP* expression in wild type, *lin*-*32*(*tm1446*), *ced*-*3*(*n717*), *ced*-*3*(*n2452*), *lin*-*32*(*tm1446*); *ced*-*3*(*n717*), *lin*-*32*(*tm1446*); *ced*-*3*(*n2452*) and *ahr*-*1*(*ia3*); *ced*-*3*(*n717*); *egl*-*13*(*ku194*) mutant animals. The *lin*-*32*(*tm1446*); *ced*-*3* double mutants partially restore *flp*-*8*::*GFP* expression in the URX neurons. In contrast, in a URX specification factor mutant background the expression of *flp*-*8*::*GFP* cannot be restored by loss of *ced*-*3*. N > 85. Note that compound loss of *ahr*-*1*(*ia3*) and *egl*-*13*(*ku194*) causes ~95% loss of *flp*-*8*::*GFP* expression^21^. (**B**) Quantification of *gcy*-*35*::*mCherry* expression in wild type, *lin*-*32*(*tm1446*), *ced*-*3*(*n717*), *ced*-*3*(*n2452*), *lin*-*32*(*tm1446*); *ced*-*3*(*n717*) and *lin*-*32*(*tm1446*); *ced*-*3*(*n2452*) mutant animals. N > 80. (**C**) Quantification of *gcy*-*36*::*GFP* expression in wild type, *lin*-*32*(*tm1446*), *ced*-*3*(*n717*), *ced*-*3*(*n2452*), *lin*-*32*(*tm1446*); *ced*-*3*(*n717*) and *lin*-*32*(*tm1446*); *ced*-*3*(*n2452*) mutant animals. N > 90. Statistical significance between wild-type and mutant strains was evaluated using one-way ANOVA analysis. ****P < 0.0001; ***P < 0.0005; **P < 0.005; n.s. not significantly different from control.

In *C*. *elegans*, the CED-3 caspase is essential for almost all apoptotic cell deaths^35^. Therefore, we analyzed the effect of *ced*-*3* loss on the URX developmental defects of *lin*-*32* mutant animals (Fig. 5A). We crossed *lin*-*32*(*tm1446*); *flp*-*8*::*GFP* animals with two independent *ced*-*3* apoptosis defective mutants - *ced*-*3*(*n717*) and *ced*-*3*(*n2452*)^36,37^. We found that loss of *ced*-*3* function partially restored the expression of *flp*-*8*::*GFP* in the URX neurons of *lin*-*32*(*tm1446*) mutant animals (Fig. 5A). Loss of *ced*-*3* was however unable to restore the expression of *flp*-*8*::*GFP* in animals lacking the URX neuron specification transcription factors, EGL-13/Sox and AHR-1/aryl hydrocarbon receptor (Fig. 5A), supporting a specific role of LIN-32 in apoptotic control. To ask whether other aspects of URX fate are restored in *lin*-*32*(*tm1446*) animals when apoptosis is suppressed, we examined two additional reporters - *gcy*-*35*::*mCherry* and *gcy*-*36*::*GFP* (Fig. 5B,C). We found that the expression of *gcy*-*36*::*GFP*, but not *gcy*-*35*::*mCherry*, was also partially restored in *lin*-*32*; *ced*-*3* double mutant animals, however at a lower degree than the *flp*-*8*::*GFP* reporter (Fig. 5A–C). This may suggest that the transcriptional requirements for driving terminal differentiation genes within the surviving URX neurons of *lin*-*32*; *ced*-*3* mutant animals are not fully intact, that additional apoptotic pathways may be inhibited, or that LIN-32 may also regulate gene expression of certain terminal fate markers.

## Discussion

This study identified the LIN-32/Atonal proneural transcription factor as a regulator of the URX neuronal lineage. We found that the expression of reporters for URX terminal fate and of URX specification genes were severely affected when LIN-32 is mutated. This disruption of URX development has an effect on organismal function, as *lin*-*32* mutant animals are unable to coordinate URX-mediated responses to changes in environmental O_2_. We further showed that loss of apoptotic caspase function promotes survival of the URX neurons in *lin*-*32* mutant animals. Taken together, our data suggest that the URX neurons, or URX precursors, undergo apoptosis in *lin*-*32* mutant animals. This finding is consistent with the function of Atonal homologs during apoptosis in other organisms^34,38^.

In other neuronal systems in *C*. *elegans*, such as the development of the male tail, the bHLH transcription factors LIN-32 and HLH-2 function together as a heterodimer^12^. We found however that HLH-2 is not required for URX development, suggesting that LIN-32 acts independently of HLH-2 in this regard, potentially in conjunction with another bHLH transcription factor. Another study showed that the HAM-1 storkhead transcription factor acts in parallel to LIN-32 to regulate Q neuroblast division^16^. We found that *ham*-*1* mutants also fail to correctly express reporters of URX neuron fate, identifying a new function for HAM-1. HAM-1 does not control URX development by promoting the expression of the serine/threonine kinase PIG-1/MELK, as it does in other *C*. *elegans* lineages^31–33^, as *pig*-*1* mutant animals express URX reporter genes.

The severe defects in the development of the URX neurons, and other neurons in the same lineage, of *lin*-*32* mutant animals suggested that either the entire sublineage is not generated or that neurons of this sublineage become hypodermal cells. However, we found that the hypodermal cell number in the head region of *lin*-*32*(*tm1446*) mutant animals is not different to wild type. We therefore hypothesized that either precursors of or the URX neurons themselves undergo inappropriate apoptosis. In support of this, we found that removal of CED-3 caspase partially restored expression of URX fate markers in the *lin*-*32* mutant but not *ahr*-*1*; *egl*-*13* double mutant animals. This is probably because AHR-1 and EGL-13 regulate gene expression in the URX neurons, whereas, LIN-32 also regulates apoptosis. Previous work has postulated that cells contain antagonistic protective and killing factors^37^ and that shifts in the balance of such factors can regulate programmed cell death decisions. Therefore, LIN-32 may be required for the regulation of this balance.

Studies from other organisms support a role for Atonal transcription factors in the regulation of apoptosis^34,38^. In *Drosophila melanogaster*, Atonal regulates apoptosis and proliferation, and overexpression of Atonal in the eye of the fly results in increased levels of caspase-3^34^. As such, Atonal in *Drosophila* promotes apoptosis in the context of the eye. Similarly, genetic studies using mouse and human colorectal cancer cells indicate a tumor suppressor function of Atoh1^39^. Here, loss of Atoh1 prevented JNK-mediated apoptosis, leading to tumor progression^39^. Both these studies therefore support a function for Atonal in the promotion of apoptosis. In contrast, studies of cochlear development in the mouse have shown that Atoh1 is required to prevent apoptosis^10^. This study showed that loss of Atoh1 leads to impaired hearing due to inappropriate apoptosis of hair cells^10^. Our findings suggest that in *C*. *elegans* LIN-32/Atonal negatively regulates apoptosis in the URX lineage. The contrasting functions for Atonal homologs in these studies in apoptotic control probably reflect the distinct cellular contexts within which Atonal acts. The identification of direct transcriptional Atonal target genes in these models will provide a better understanding of how this conserved transcription factor regulates cell survival. Intriguingly, Atoh1 is expressed in the dorsal hindbrain of mammals and loss of Atoh1 results in respiratory failure in mice before birth^40^. Therefore, our finding that LIN-32/Atonal is required for correct development of O_2_-sensing neurons in *C*. *elegans* may have revealed a conserved function for Atonal homologs.

## Methods

### Mutant and transgenic reporter strains

Worms were cultured using standard conditions on NGM agar plates and maintained at 20 °C^41^. A complete list of strains used for this study is detailed in Table S1.

### Forward genetic screen

In the forward genetic screen, the URX reporter strain *ynIs22*[*flp*-*8*::*GFP*] was mutagenized with EMS (ethyl methanesulfonate) according to standard protocols^41^. Potential URX cell fate mutants in the F2 population were manually isolated based on loss of GFP signal under a dissecting fluorescence stereomicroscope.

### Mapping of *rp1*

The genomic lesion of *rp1* was identified using single-nucleotide polymorphism (SNP) mapping^24^. Hawaiian (CB4856) males were crossed into *rp1*; *flp*-*8*::*GFP* hermaphrodites and the mutant phenotype (URX loss) was recovered in the F2 generation. The progeny from 10 F2’s were examined by SNP mapping and the causative lesion was linked to the genetic positions of -17 and -8 on the X chromosome. Subsequent single worm SNP mapping showed strong linkage to -17. The inability of *rp1* males to mate and the loss of PVM expression of *flp*-*8*::*GFP*, two previously identified phenotypes of *lin*-*32* mutant animals^15,42^, suggested that there was a lesion in *lin*-*32* locus. Sequencing of *rp1* revealed a missense mutation at position 376 of the *lin*-*32* cDNA, leading to a premature STOP amber codon (Q125STOP).

### Fluorescence microscopy

L4/young adult animals were analyzed for neuronal cell fate defects by mounting them on a glass slide with a 5% agarose pad, using 20 mM NaN_3_ as an anesthetic. Images were taken using an upright fluorescence microscope (Zeiss, AXIO Imager M2) and ZEN software (version 2.0).

### Behavioral assays

Oxygen sensing behavioral assays were conducted as previously described^18,21,27^. Wild type and *rp1* mutant animals were starved for 1 h and then transferred to 14-cm NGM plates containing a 56 × 56 mm arena of Whatman filter paper soaked in 20 mM CuCl_2_. Between 80 and 120 animals were used in a single experiment and each experimental condition was repeated four to six times. A custom-made transparent plexiglass chamber with a flow volume of 60 × 60 × 0.7 mm was placed onto the assay arena and animals were accustomed to a gas flow of 100 ml/min containing 21% (v/v) O_2_ for 5 min. Animals were stimulated for 6 min with 10% O_2_ and 0% CO_2_. In all conditions, the gas compositions were balanced with N_2_. Gases were mixed by red-y gas mixing units (Vögtlin Instruments) and controlled by LabView software. Recordings were illuminated with flat red LED lights and made at three frames per second on a 4-megapixel CCD camera (Jai), using Streampix software (Norpix). For movie analysis, MatLab-based image processing and tracking scripts were used as previously described^43,44^. The resulting trajectories were used to calculate instantaneous speed during continuous forward movements (1-sec binning).

### Statistical analysis

Statistical analyses were performed in Graphpad Prism 7 using t-test or one-way ANOVA where applicable. Differences with a P-value < 0.05 were considered significant.

## Electronic supplementary material

Supplementary Information
