# Supplementary material for: LIN-32/Atonal Controls Oxygen Sensing Neuron Development in Caenorhabditis elegans
Source: Sci Rep. 2017 Aug 4;7:7294. doi: 10.1038/s41598-017-07876-4 (PMC5544745; doi:10.1038/s41598-017-07876-4)
Supplement: Supplementary file 1 — Supplementary Information [file 41598_2017_7876_MOESM1_ESM.pdf]

LIN-32/Atonal Controls Oxygen Sensing Neuron Development in *Caenorhabditis elegans*

Teresa Rojo Romanos[1,2], David Pladevall-Morera[1,2], Kasper Langebeck-Jensen[2], Stine Lind Hansen[2], Leelee Ng[1] and Roger Pocock[1,2].

Table S1.

List of strains used in this study.

	Strain Number	Genotype
Figure 1	RJP206	*ynIs22[flp-8::GFP]*
	RJP30	*lin-32(rp1); ynIs22[flp-8::GFP]*
	RJP36	*lin-32(tm1446): ynIs22[flp-8::GFP]*
	RJP1816	*lin-32(tm1446): ynIs22[flp-8::GFP]; ezIs10[LIN-32::GFP]*
Figure 2	RJP255	*ynIs34[flp-19::GFP]; him-5(e1490)*
	RJP61	*lin-32(tm1446); ynIs34[flp-19::GFP]*
	CX8600	*kyIs417[odr-1::dsRed, gcy-36::GFP]*
	RJP3169	*lin-32(tm1446); kyIs417[gcy-36::GFP, odr-1::dsRed]*
	RJP1264	*rpEx588[gcy-35::mcherry]*
	RJP3192	*lin-32(tm1446); rpEx588[gcy-35::mcherry]*
	RJP1458	*rpIs32[egl-13::GFP]*
	RJP1759	*lin-32(tm1446); rpIs32[egl-13::GFP]*
	RJP771	*rpEx379[punc-86(2.5kb)::GFP]*
	RJP1758	*lin-32(tm1446); rpEx379[punc-86(2.5kb)::GFP]*
	-	*N2*
	RJP3292	*lin-32(tm1446)*
Figure 3	BY200	*vtIs1[dat-1::GFP,rol-6]*
	RJP1754	*lin-32(tm1446); vtIs1[dat-1::GFP,rol-6]*
	RJP29	*ynIs80[flp-21::GFP]*
	RJP1752	*lin-32(tm1446); ynIs80[flp-21::GFP]*
Figure 4	RJP3227	*ham-1(n1438); kyIs417[gcy-36::GFP, odr-1::dsRed]*
	RJP3278	*ham-1(n1438); lin-32(tm1446); kyIs417[gcy-36::GFP, odr-1::dsRed]*
	RJP1846	*ham-1(n1438); ynIs22[flp-8::GFP]*
	RJP3243	*ham-1(n1438); lin-32(tm1446); ynIs22[flp-8::GFP]*
	RJP3260	*pig-1(gm344); kyIs417[gcy-36::GFP, odr-1::dsRed]*
	RJP3260	*pig-1(gm344); lin-32(tm1446); kyIs417[gcy-36::GFP, odr-1::dsRed]*
	RJP1970	*pig-1(gm344); ynIs22[flp-8::GFP]*
	RJP3270	*pig-1(gm344); lin-32(tm1446); ynIs22[flp-8::GFP]*
	RJP3258	*hlh-2(tm1768); kyIs417[gcy-36::GFP, odr-1::dsRed]*
	RJP3286	*hlh-2(tm1768); lin-32(tm1446); kyIs417[gcy-36::GFP, odr-1::dsRed]*
	RJP430	*hlh-2(tm1768); ynIs22[flp-8::GFP]*
	RJP3276	*hlh-2(tm1768); lin-32(tm1446); ynIs22[flp-8::GFP]*
Figure 5	RJP2029	*ced-3(n717) unc-26(e205); ynIs22[flp-8::GFP]*
	RJP1954	*ced-3(n717) unc-26(e205); lin-32(tm1446); ynIs22[flp-8::GFP]*
	RJP3126	*ced-3(n2452); ynIs22[flp-8::GFP]*
	RJP3125	*ced-3(n2452); lin-32(tm1446); ynIs22[flp-8::GFP]*
	RJP3110	*ahr-1(ia3); ced-3(n717) unc-26(e205); egl-13(ku194); ynIs22[flp-8::GFP]*
	RJP3191	*ced-3(n717); rpEx588[gcy-35::mcherry]*
	RJP3207	*ced-3(n717); lin-32(tm1446); rpEx588[gcy-35::mcherry]*
	RJP3197	*ced-3(n2425); rpEx588[gcy-35::mcherry]*
	RJP3194	*ced-3(n2452); lin-32(tm4146); rpEx588[gcy-35::mcherry]*
	RJP3171	*ced-3(n717); kyIs417[gcy-36::GFP, odr-1::dsRed]*
	RJP3174	*ced-3(n717); lin-32(tm1446); kyIs417[gcy-36::GFP, odr-1::dsRed]*
	RJP3170	*ced-3(n2452); kyIs417[gcy-36::GFP, odr-1::dsRed]*
	RJP3173	*ced-3(n2452); lin-32(tm1446); kyIs417[gcy-36::GFP, odr-1::dsRed]*
Data in text	RJP3221	*rpIs109[dpy-7::NLS::dsRed2]*
	RJP1847	*lin-32(tm1446); rpIs109[dpy-7::NLS::dsRed2]*
